# Description and evaluation of experimental models for uterine transplantation in pigs

**DOI:** 10.1590/S1679-45082017AO4066

**Published:** 2017

**Authors:** Emerson de Oliveira, Kelly Alessandra da Silva Tavares, Mariano Tamura Vieira Gomes, Alcides Augusto Salzedas-Netto, Marair Gracio Ferreira Sartori, Rodrigo Aquino Castro, César Eduardo Fernandes, Manoel João Batista Castello Girão

**Affiliations:** 1 Faculdade de Medicina do ABC, Santo André, SP, Brazil; 2 Hospital Israelita Albert Einstein, São Paulo, SP, Brazil; 3 Escola Paulista de Medicina, Universidade Federal de São Paulo, São Paulo, SP, Brazil

**Keywords:** Transplantation, Uterus/transplantation, Infertility, Pregnancy, Swine, Models, animal, Immunosuppressive agents, Cyclosporine, Transplante, Útero/transplante, Infertilidade, Gravidez, Suínos, Modelos animais, Imunossupressores, Ciclosporina

## Abstract

**Objective:**

To evaluate the technique of uterine transplantation and the use of drugs used in the process of immunosuppression.

**Methods:**

We included 12 sows, and immunosuppression was performed with minimal doses of cyclosporine, and cross-match was done to exclude the possibility of blood incompatibility. Hysterectomy was performed in the donor under general anesthesia, with the removal of the aorta and inferior vena cava in monobloc, and anastomosis of these vessels was made in the recipient.

**Results:**

Six experiments were performed, and on the immediate postoperative period, five animals had good reperfusion. However, on the seventh postoperative day, histological analysis showed rejection in five animals.

**Conclusion:**

The experimental model of uterine transplantation is feasible, but monitoring doses of immunosuppressants is pivotal to prevent rejection episodes.

## RESUMO

**Objetivo:**

Avaliar a técnica de transplante uterino e o uso de drogas no processo de imunossupressão.

**Métodos:**

Foram incluídas 12 porcas, sendo realizada imunossupressão com doses mínimas de ciclosporina, e prova cruzada para afastar a possibilidade de incompatibilidade sanguínea. Realizou-se, na doadora, histerectomia sob anestesia geral, com a retirada, em monobloco, da aorta e da veia cava inferior, de tal forma que, na receptora, fosse possível realizar a anastomose com estes vasos.

**Resultados:**

Foram realizados seis experimentos e, no pós-operatório imediato, houve boa reperfusão em cinco animais. Entretanto, no sétimo dia de pós-operatório, as análises histológicas demonstraram rejeição em cinco deles.

**Conclusão:**

O modelo experimental de transplante uterino é factível, mas a monitorização das doses de imunossupressores é importante, a fim de impedir os episódios de rejeição.

## INTRODUCTION

Recent advancements of Reproductive Medicine, especially in some technical aspects, such as hormonal stimulation, *in vitro* fertilization (IVF), and the intracytoplasmic sperm injection (ICSI), made it possible to solve many causes of male and female infertility.^(^ ^1^ ^)^ For those women with absolute infertility, the traditional options of maternity are adoption or surrogate pregnancy.^(^ ^2^ ^)^

However, in many countries, surrogate pregnancy is not accepted due to legal, ethical and religious issues.^(^ ^3^ ^)^ Thus, women submitted to hysterectomy when young due to malignant gynecological diseases or benign conditions, such as leiomyoma, endometriosis, and adenomyosis; patients with significant blood loss after a delivery resulting in hysterectomy, and finally, women with congenital anomalies of the genital tract, such as the Mayer-Rokitansky-Küster-Hauser syndrome, are condemned to not bearing children. Unquestionably, for many women this perspective affects their quality of life very negatively.^(^ ^4^ ^–^ ^7^ ^)^

The diverse issues related to surrogate pregnancy have led these women to ‘dream’ of the possibility of a uterine transplant that would relieve the anguish of the immense desire to conceive and bear a child.^(^ ^8^ ^)^

Uterine transplant mimicks a normal situation, with the primary constituent of genetic, gestational, and legal maternity. Additionally, the usual health risks associated with pregnancy, such as thromboembolism, hypertension, diabetes, and delivery complications, would be related to the genetic mother, and not to the surrogate mother.^(^ ^2^ ^)^

Over the last years, many advances in organ transplants have been reached. Techniques of microvascular anastomosis and antirejection drugs are becoming safe.^(^ ^9^ ^)^ Transplantation of non-vital organs, like face and hands, has been reported.^(^ ^10^ ^)^ Nevertheless, despite the advances mentioned, uterine transplant is still a controversial topic.^(^ ^9^ ^)^

In 2009, the Committee for the Ethical Aspects of Human Reproduction and Women's Health of the International Federation of Gynecology and Obstetrics (FIGO) prepared a document that established the guidelines for uterine transplants in humans. According to FIGO, considering the prevalence of female infertility due to uterine factors of about 3 to 5% and the issues that involve surrogate gestation, in these specific situations a transplant is justified.^(^ ^11^ ^)^ However, any procedure in humans would only be justified after trials carried out in adequate animal models, which should include primates, due to the similarity of anatomical structures with those of the human species.^(^ ^11^ ^)^

## OBJECTIVE

To evaluate the technique of uterine transplant and the drugs used in the process of immunosuppression in an animal model.

## METHODS

This study was performed by means of a partnership between the *Universidade Federal de São Paulo* (UNIFESP), registered and approved by the Research Ethics Committee under number 2112/08, and the *Instituto Israelita de Ensino e Pesquisa Albert Einstein* (IIEP), registered and approved by Research Ethics Committee under number CEUA 1074-09. During the period between April 2009 and April 2011, six experiments were performed (12 operations) at the Surgical Experimentation and Training Center (CETEC - *Centro de Experimentação e Treinamento em Cirurgia* ) of *Hospital Israelita Albert Einstein* (HIAE). All activities at CETEC followed the ethical procedures and the best practices in treatment and in their use in animals.

Twelve Large White sows, aged 8-13 months and weight varying between 30 and 40kg were included. Crossmatch was performed to rule out the possibility of blood incompatibility of the animals - donors and recipients.

### Procedures in the donor and in the recipient

The anesthesia protocol for swine consists of solid and fluid fasting for approximately 12 hours. In the bays, the animals received the pre-anesthetic medications: ketamine hydrochloride in bolus of 10mg/kg associated with midazolam 0.25mg/kg, via intramuscular injection in the buttocks. After five minutes, the sows were showered to remove dirt and debris from the body; then they were weighed and sent to the operating room ( Table 1 ).

**Table 1:** Weight of donors and recipients

Experiment	Donor weight (kg)	Recipient weight (kg)
1	29.6	33.5
2	28	29
3	30	36
4	25	28
5	26	28
6	27	24

The peripheral veins of the animals were punctured with Jelco 22, and anesthetic induction was done with etomidate (1 to 2mg/kg=15mL). After relaxation as soon as the eyes showed rotation, the animals were intubated with a 6.5 orotracheal tube. Anesthesia was maintained with 1.5% isoflurane gas diluted in 2 liters of oxygen, with a tidal volume of 10mL/kg/hour. Before beginning the surgical procedure, analgesics and muscle relaxants were applied. Analgesic administration was done with fentanyl 2.5mcg/kg, and the muscle relaxants used were cisatracurium besylate, at 0.24mg/kg, and pancuronium at 0.2mg/kg.

Electrodes and an oximeter were placed on the animals to monitor them during the entire procedure. Asepsis and antisepsis were done on the abdominal and inguinal regions.

Immunosuppression was performed as shown on table 2 .

**Table 2:** Dose of cyclosporine administered per day

Day	Event	Route	Dose (mg/kg/day)
D1	Transoperative	Intravenous	2
D2	Transoperative	Intravenous	2
D3	Postoperative	Oral	5
D4	Postoperative	Oral	5
D5	Postoperative	Oral	5
D6	Postoperative	Oral	5
D7	Postoperative	Oral	5
D8	Postoperative	Oral	5
D9	Postoperative	Oral	5
D10	Postoperative	Oral	5
D11	Postoperative	Oral	5
D12	Postoperative	Oral	5
D13	Postoperative	Oral	5
D14	Postoperative	Oral	5

The donor and recipient animals were submitted to the following procedures: asepsis of the skin with a degerming agent, povidone-iodine (PVP-I), positioning of the sterile surgical drapes, median xipho-pubic incision of the skin, opening of the subcutaneous tissue, and hemostasis; opening of aponeurosis, visualization of abdominal cavity, inspection of organs, and dissection of the peritoneum until identification of the aorta and inferior vena cava.

**Figure 1:**
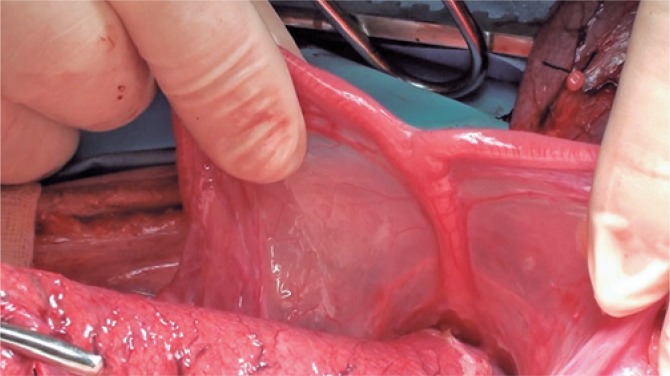
Donor uterus

**Figure 2:**
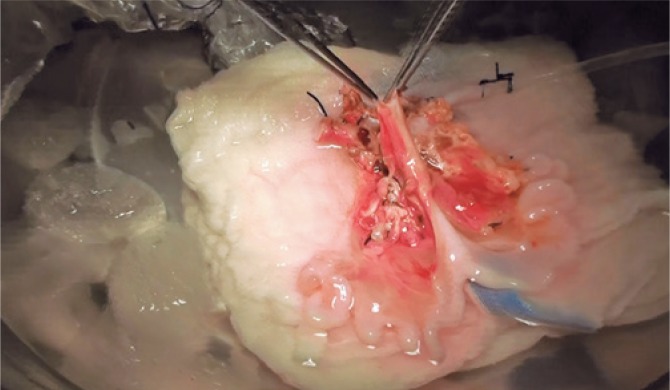
Final aspect of the transplanted heterotopic uterus

In the donor, the following technique was used: ligation of the dorsal branches of the aorta with cotton 3.0; dissection of the retroperitoneum until identification of the external iliac arteries bilaterally; dissection of the broad ligament until isolation of the uterine horn, ligation of the ureter at the insertion of the bladder, caudal dissection of the internal iliac artery until the origin of the uterine artery, followed by ligation of the iliac vessels, from the vaginal branch of the internal iliac artery bilaterally, of the versicouterine ligament and uterine vessels bilaterally with cotton 2.0, and of the pelvic infundibular ligaments; preparation of a solution with 0.9% saline at room temperature and 0.9% frozen saline, maintaining the solution at 4°C; catheterization of the external iliac artery with temporary urinary catheter number 14; ligation of the aorta; infusion of the Collins solution through the catheter of the external iliac artery until complete cleansing of the circulation to the uterus; sectioning of the inferior vena cava to euthanize the animal; ligation of the external iliac artery bilaterally; sectioning of the vagina at the uterine cervix; removal of the uterus and its preservation in the frozen saline; and closure of the donor cavity with Vicryl-0 in continuous suture ( Figures 1 and 2 ).

**Figure 3:**
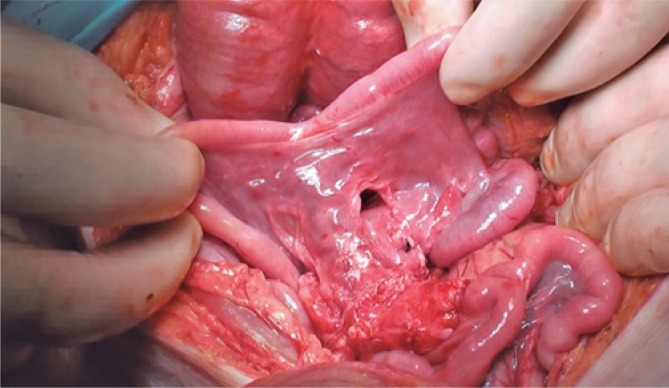
Final aspect of the transplanted heterotopic uterus

The recipient was submitted to the following technique: repair of the proximal and distal vessels with the aid of cotton sutures 2.0, and grasping the inferior vena cava using the Satinsky vascular clamps, followed by its sectioning. Later, termino-lateral anastomosis was performed with the vena cava from the graft, and continuously sutured with Prolene 7.0 and Satinsky clamps. The aorta was grasped and then sectioned, and termino-lateral anastomosis was made with the aorta from the graft, and continuously sutured with Prolene 7.0. Reperfusion and hemostatic review of the graft were done, with anastomosis of the donor's vaginal cavity and the recipient using Vicryl 4.0, synthesis of the aponeurosis with Vicryl 1.0, and synthesis of the skin with Vicryl 1.0 using interrupted sutures ( Table 3 and Figure 3 ).

**Table 3:** Control of surgery in the recipient

Experiment	Start of anesthesia	Start of surgery	Clamping	Start of anastomosis	Reperfusion	Heparinization	End of procedure
1	12:40pm	12:50pm	1:00pm	1:05pm	1:45pm	1:45pm	2:15pm
2	1:20pm	1:35pm	2:00pm	2:05pm	3:10pm	3:10pm	4:30pm
3	1:40pm	1:50pm	2:00pm	2:05pm	2:45pm	2:45pm	3:15pm
4	11:40am	11:50am	noon	12:05pm	12:45pm	12:45pm	1:15pm
5	noon	12:24pm	1:00pm	1:05pm	1:45pm	1:45pm	2:50pm
6	12:40pm	12:50pm	1:00pm	1:05pm	1:45pm	1:45pm	2:15pm

## RESULTS

After reanastomosis, vitality of the graft (reperfusion) was evaluated. In five recipients, except in the animal of the fourth experiment, the recipients were followed up daily, for seven days. On the seventh postoperative day, the five animals were sent for laparotomy, which followed the same anesthetic care already described. After opening the cavity, necrosis of the implanted uterus was observed. At this time, the implanted organ was collected and the animals were euthanized, and then sent to the freezer for posterior incineration. In the animal of the fourth experiment, after reanastomosis, the absence of graft vitality was observed (poor reperfusion). Next, the implanted organ was collected and the animal was euthanized and sent to the freezer for posterior incineration.

Histological evaluations of the implanted uteri were made. In the first experiment, necrosis was demonstrated by a likely mechanical component and the absence of signs of rejection. In the second, third, fifth, and sixth experiments, necrosis was demonstrated by acute rejection (presence of inflammatory infiltrate with glandular aggression and capillaritis, especially by neutrophils). Necrosis by hyperacute rejection (presence of inflammatory infiltrate with extreme glandular damage and capillaritis, especially by neutrophils) was noted in the animal of the fourth experiment ( Chart 1 ).

**Chart 1:** Histological aspect of the uterus

Experiment	Vitality of the graft	Histological aspect
1	Excelente	Absence of rejection
2	Excelente	Acute rejection
3	Excelente	Acute rejection
4	Ausente	Hyperacute rejection
5	Excelente	Acute rejection
6	Excelente	Acute rejection

## DISCUSSION

The first human uterine transplant was performed in Saudi Arabia, in 2000. However, the transplanted uterus remained in the recipient for 99 days, when occlusion of the uterine vessels occurred due to thrombosis.^(^ ^8^ ^)^ In Turkey, in 2011, a 21-year-old patient born with no a uterus received the first uterine transplant from a deceased donor. The preliminary results of this procedure were published in 2013 and did not reveal rejection to the graft.^(^ ^12^ ^)^

The Swedish group of Dr. Mats Bräannström has a line of research on uterine transplants in live donors. This group performed nine uterine transplants and did not report any immediate postoperative complications. It was recorded that, after six months, seven patients had a viable graft, and two lost the graft due to thrombotic phenomena.^(^ ^13^ ^)^ The group considers that, unlike any other type of transplant, the uterine graft is transient, that is, it is removed as soon as the desired result is reached, *i.e* ., one or two healthy gestations. Thus, immunosuppression is done for a limited period. In their studies with animal models, the members of the group assured that, the procedure is compatible with fully normal gestations, even when women are on immunosuppression.^(^ ^13^ ^)^

In our research, we used a minimal dose of cyclosporine, and this probably led to a high frequency of rejection. Additionally, we did not control the serum levels of the immunosuppressant, which might have had an expressive negative contribution in this aspect. Our results differ from those of the project by Avison et al., who described the first model of uterine transplant in swine and concluded that such model is feasible in heterotopic transplants.^(^ ^14^ ^)^ Immunosuppression was carried out with intravenous tacrolimus during the first 12 days after transplant, followed by maintenance immunosuppression with oral cyclosporine. Acute graft rejections during the second and third post-transplant months were treated successfully with increased maintenance immunosuppression and steroids.^(^ ^14^ ^)^

Unlike other organs supplied by large-diameter vessels, the uterus receives blood from a network of small-diameter vessels. This means that establishing blood flow to a transplanted organ is extremely complex and prone to problems. We began our project with the model in rabbits. However, right away in the first procedures we identified the need for a change in animal model. Rabbits are stressed and fragile animals, with very small blood vessels. Therefore, we chose to work with a swine model. Additionally, vessels that supply blood to the uterus should be able to expand up to three times during gestation in order to sustain the development of the fetus.

## CONCLUSION

Unquestionably, our work is an arduous and complex task. In Brazil, it is a pioneer project. We were successful in performing the operative technique, but realized that it is necessary to have rigorous observation and control of immunosuppressant levels. Our results suggest that drugs that are more modern should be used in the process of immunosuppression. Further studies should be conducted, so that there is progress in this fascinating field of knowledge, which can improve the quality of life of many women.
